# Emerging zoonotic ocular sporotrichosis in southeast Asia: a case series from Thailand and systematic review of regional reports

**DOI:** 10.1186/s12348-025-00565-8

**Published:** 2026-02-24

**Authors:** Usanee Reinprayoon, Trakanta Wannapanich, Buravej Assavapongpaiboon, Ngamjit Kasetsuwan, Thanachaporn Kittipibul, Suppapong Tirakunwichcha, Ariya Chindamporn, Nattapong Langsiri

**Affiliations:** 1 Center of Excellence for Cornea and Stem Cell Transplantation, Department of Ophthalmology, Faculty of Medicine, Chulalongkorn University, Bangkok, Thailand; 2 Department of Ophthalmology, Faculty of Medicine, Chulalongkorn University and King Chulalongkorn Memorial Hospital, 1873 Rama IV Road, Pathum Wan, Bangkok, 10330 Thailand; 3 Excellence Center for Cornea and Stem Cell Transplantation, Department of Ophthalmology, King Chulalongkorn Memorial Hospital, Thai Red Cross Society, Bangkok, Thailand; 4 Department of Microbiology, Faculty of Medicine, Chulalongkorn University, Bangkok, Thailand; 5 Department of Transfusion Medicine and Clinical Microbiology, Faculty of Allied Health Science, Chulalongkorn University, Bangkok, Thailand; 6 Center of Excellence of Medical Mycology Diagnosis, Chulalongkorn University, Bangkok, Thailand; 7 Center of Excellence in Antimicrobial Resistance and Stewardship, Department of Microbiology, Faculty of Medicine, Chulalongkorn University, Bangkok, Thailand

**Keywords:** Sporotrichosis, Eye infections, Conjunctivitis, Mycoses, Zoonoses

## Abstract

**Background:**

To describe the clinical features, diagnostic approaches, and treatment outcomes of ocular sporotrichosis through an institutional case series and systematic review of reports from Southeast Asia.

**Methods:**

Five patients diagnosed with ocular sporotrichosis at a tertiary referral center in Thailand (2020–2024) were retrospectively reviewed for clinical presentation, diagnostic confirmation, and treatment outcomes. A systematic review of PubMed, Embase, Scopus, and MEDLINE (last searched March 20, 2025) identified published cases from Southeast Asia. Eligible reports were narratively synthesized; no meta-analysis was performed due to clinical heterogeneity.

**Results:**

All five institutional cases presented with chronic granulomatous conjunctivitis, frequently associated with cat exposure. Diagnosis was confirmed by fungal culture and/or histopathology in all patients. Oral itraconazole was prescribed in every case, with adjunctive topical antifungals used selectively. Most patients improved clinically, though several were lost to follow-up. The systematic review included 12 studies comprising 23 patients reported between 2018 and 2024. The median age was 32 years (IQR 22.5–55.0), and 78.3% were female. Most cases were unilateral (91.3%), with microbiological confirmation in 87%. Oral itraconazole was the primary treatment, with topical or intralesional antifungals in some cases. Clinical outcomes were generally favorable, though one patient developed limbal stem cell deficiency.

**Conclusions:**

Ocular sporotrichosis is an emerging zoonotic infection in Southeast Asia, often linked to cat exposure. Early recognition and prompt antifungal therapy are critical to achieve good outcomes and prevent sight-threatening complications.

**Supplementary Information:**

The online version contains supplementary material available at 10.1186/s12348-025-00565-8.

## Background

*Sporothrix* spp. is a thermally dimorphic fungus found worldwide, primarily in soil and decaying organic matter. It infects humans and animals, causing sporotrichosis, a chronic fungal infection that most often presents with cutaneous and subcutaneous lesions. The disease is often linked to exposure to plants, soil, or animals [1]. Of more than 50 known species, *S. brasiliensis*, *S. schenckii*, and *S. globosa* are the main pathogens in clinical settings. *S. brasiliensis*, known for its high virulence, is associated with zoonotic outbreaks in Brazil and Argentina. In contrast, *S. schenckii* and *S. globosa* are globally distributed and usually environmentally transmitted [1]. Clinical manifestations range from lymphocutaneous and fixed cutaneous forms to disseminated disease, particularly in immunocompromised individuals [1, 2]. 

Ocular sporotrichosis is a less common but increasingly recognised manifestation. It typically results from the implantation of the filamentous (mould) form of *Sporothrix* into the ocular surface. The primary causative species include *S. brasiliensis*, *S. schenckii*, *S. globosa*, and *S. luriei*. Reports of *S. brasiliensis* have largely originated from South America, while *S. schenckii* complex have recently been identified in Southeast Asia, including Thailand and Malaysia. While conventional microscopy allows genus-level identification based on characteristic fungal morphology, species- and clade-level classification requires molecular techniques such as polymerase chain reaction (PCR) and internal transcribed spacer (ITS) sequencing [3–5]. 

Clinically, ocular sporotrichosis most often presents as granulomatous conjunctivitis involving the tarsal conjunctiva, with yellowish nodules that facilitate recognition. Other ocular manifestations include acute dacryocystitis and Parinaud oculoglandular syndrome, characterised by granulomatous conjunctivitis and regional lymphadenopathy. Importantly, sporotrichosis-associated conjunctivitis may mimic other forms of conjunctivitis but often includes purulent discharge, aiding in differentiation [2]. 

Globally, the distribution of ocular sporotrichosis varies by anatomic form and geography: eyelid cases predominate in China and Peru, conjunctival cases in hyperendemic Brazil, and intraocular disease mainly in the United States and Brazil [6]. These global patterns contextualise the current series from Southeast Asia, where documented cases remain limited. In Thailand, ocular sporotrichosis is an emerging zoonosis [3]. The increasing number of cases is partly attributed to transmission from infected cats, which act as reservoirs of *Sporothrix* spp. Infection can occur through respiratory secretions during close contact, such as sneezing [2, 3, 7, 8]. Increasing awareness among ophthalmologists and healthcare providers is essential, as ocular sporotrichosis can mimic more common ocular surface diseases.

This study aims to address the growing importance of ocular sporotrichosis in Thailand and Southeast Asia by presenting a case series from a tertiary care centre and systematically reviewing existing literature. The findings are intended to enhance recognition, facilitate timely diagnosis, and guide management of this underdiagnosed but clinically significant infection.

## Materials and methods

### Institutional case series

This retrospective case series included five patients diagnosed with ocular sporotrichosis at the Department of Ophthalmology, King Chulalongkorn Memorial Hospital, Bangkok, Thailand. Medical records between September 2020 and December 2024 were reviewed. The study was approved by the institutional review board (IRB No.0903/67, COA No. 0084/2025). The requirement for informed consent was waived by the IRB due to the retrospective nature of the study and all clinical photographs have been de-identified. Patients were included if they had clinical features suggestive of ocular sporotrichosis and either:

(1) A microbiologically confirmed diagnosis (positive fungal culture for *Sporothrix schenckii* complex from conjunctival swab or biopsy), or.

(2) A presumed clinical diagnosis based on characteristic ocular findings and compatible exposure history, in cases where microbiological tests were negative (“suspected case”).

Data were retrieved from electronic medical records, including demographics, risk factors (e.g., animal contact, trauma), ocular and extraocular findings, microbiological and histopathological investigations, treatment modalities (topical and systemic antifungal agents), treatment duration, and outcomes. One patient was categorized as “suspected case” based on suggestive ocular features and supportive histopathology despite negative fungal culture.

Descriptive statistics were used to summarise demographic characteristics, clinical presentations, treatment regimens, and outcomes. Continuous variables such as age and treatment duration were reported as medians and interquartile ranges (IQR), while categorical variables were summarised using frequencies and percentages.

### Systematic review

This systematic review was conducted according to the PRISMA 2020 guidelines [9] and aimed to investigate the clinical spectrum, diagnostic strategies, treatment approaches, and associated risk factors of ocular sporotrichosis reported in Southeast Asia. The review was not preregistered in a public repository because it primarily synthesized rare case reports and small case series, which do not meet eligibility criteria for platforms such as PROSPERO.

### Review question

What are the clinical characteristics, diagnostic methods, management approaches, and risk factors associated with ocular sporotrichosis reported in Southeast Asian countries?

### Eligibility criteria

Studies were included if they described at least one human case of ocular sporotrichosis originating from Southeast Asian countries. Animal studies, laboratory experiments, and non-original publications such as reviews or editorials were excluded. The detailed inclusion and exclusion criteria are provided in Supplementary Table S1.

### Information sources and search strategy

A comprehensive search was performed in PubMed, Embase, Scopus, and MEDLINE via Ovid from database inception to March 20, 2025, using combinations of MeSH terms and keywords related to “ocular sporotrichosis”, “*Sporothrix*”, and related anatomical or clinical descriptors. Boolean operators were applied to maximize retrieval sensitivity. To enhance regional coverage, supplementary searches were conducted in ThaiJO and Google Scholar, which yielded no additional eligible studies. The complete search strategy is provided in Supplementary Table S2.

### Study selection

**Fig. 1:**
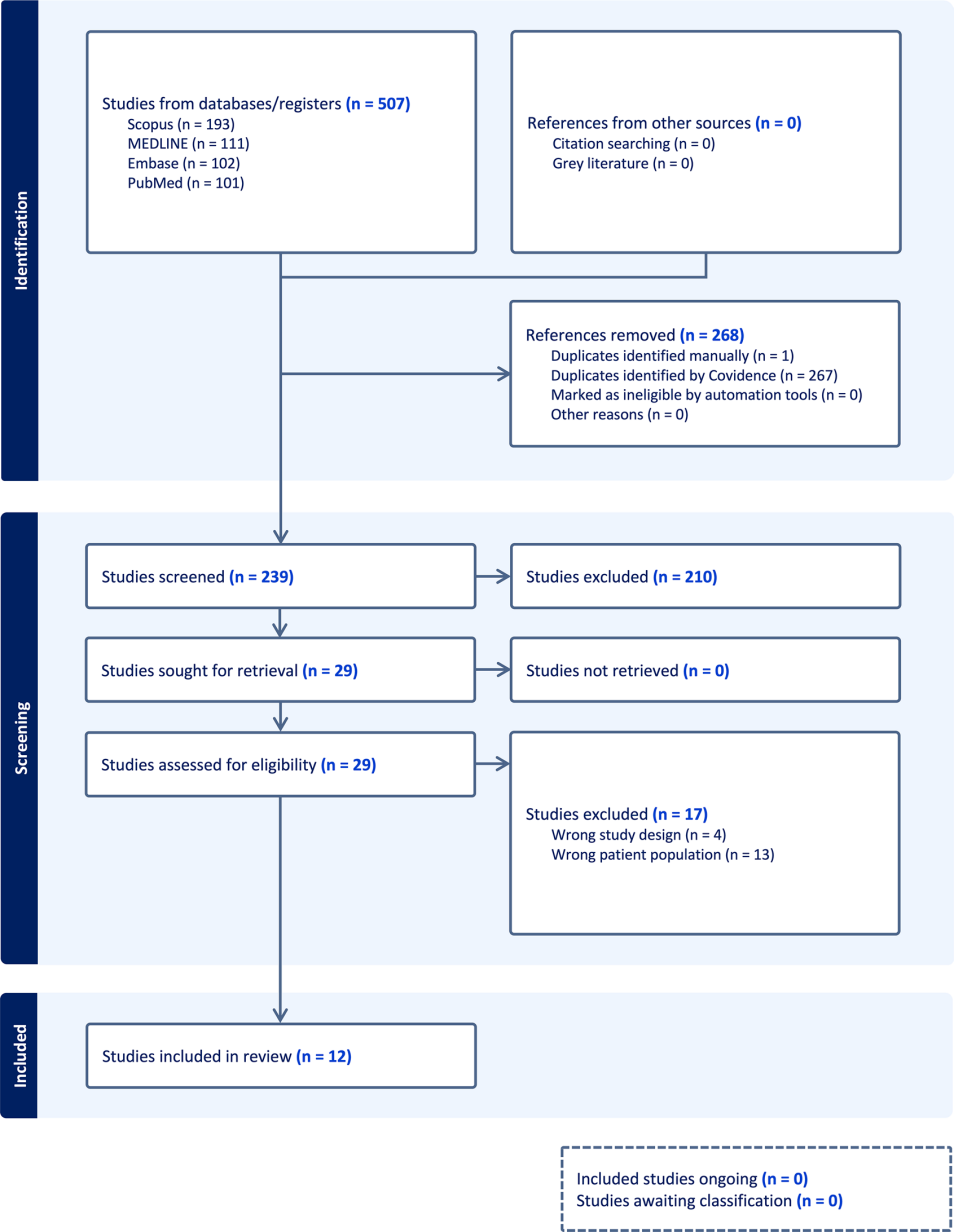
PRISMA 2020 Flow diagram of the study selection process for the systematic review on ocular sporotrichosis. [Diagram generated using Covidence systematic review software (www.covidence.org).]

Search results were imported into Covidence systematic review software (Veritas Health Innovation, Melbourne, Australia; www.covidence.org) for screening and data management. Two independent reviewers (TW and UR) screened titles, abstracts, and full texts. Disagreements were resolved through discussion with a third reviewer (NK). Non-English full-text articles were included when available and translated into English using Google Translate, with translations reviewed by the authors for accuracy. The PRISMA 2020 flow diagram is shown in Fig. 1.

### Data extraction and analysis

Data were extracted manually into a standardised form designed for this review. One reviewer (TW) extracted, and another (UR) verified the data. For each included study, data were extracted on patient demographics, exposure history, clinical presentation, diagnostic methods, treatment, and outcomes. Each case was analysed individually. Data synthesis was descriptive due to the small number and heterogeneity of included studies.

### Synthesis methods

Given the small number and heterogeneity of cases, data were summarised narratively by key domains, including clinical features, diagnostics, treatment, and outcomes. Structured summary tables were used for comparison across cases. No meta-analysis or subgroup analyses were performed due to the descriptive nature of the data and lack of comparable outcome metrics.

### Quality assessment

The methodological quality of included studies was evaluated using the Joanna Briggs Institute (JBI) Critical Appraisal Checklists for Case Reports and Case Series [10]. Two reviewers (TW and UR) independently appraised each study and discrepancies were resolved through consensus. No studies were excluded based on quality, as the aim was to provide a comprehensive descriptive overview.

### Reporting bias assessment

Due to the limited number and nature of included studies (primarily case reports and small case series), a formal assessment of reporting bias or publication bias was not performed. The potential for publication bias is acknowledged as a limitation.

### Certainty assessment

The certainty of the body of evidence was not formally assessed using GRADE or other frameworks. This decision was based on the descriptive nature and small sample size of included reports, which did not permit reliable evaluation of evidence certainty.

ChatGPT (GPT-5, OpenAI, San Francisco, USA) was used to refine grammar and language during manuscript preparation. All generated content was reviewed and verified by the authors to ensure accuracy and compliance with scientific and ethical standards. No generative AI tools were used for data analysis, interpretation, or drawing scientific conclusions.

## Results

### Institutional case series

Five patients diagnosed with ocular sporotrichosis at King Chulalongkorn Memorial Hospital were retrospectively reviewed. Their demographic and clinical characteristics, risk factors, microbiological investigations, treatment regimens, and outcomes are described below. One additional culture-negative case with compatible clinical and epidemiologic features is presented separately as *a suspected ocular sporotrichosis case* (Supplementary Appendix 1).

### Case 1

**Fig. 2:**
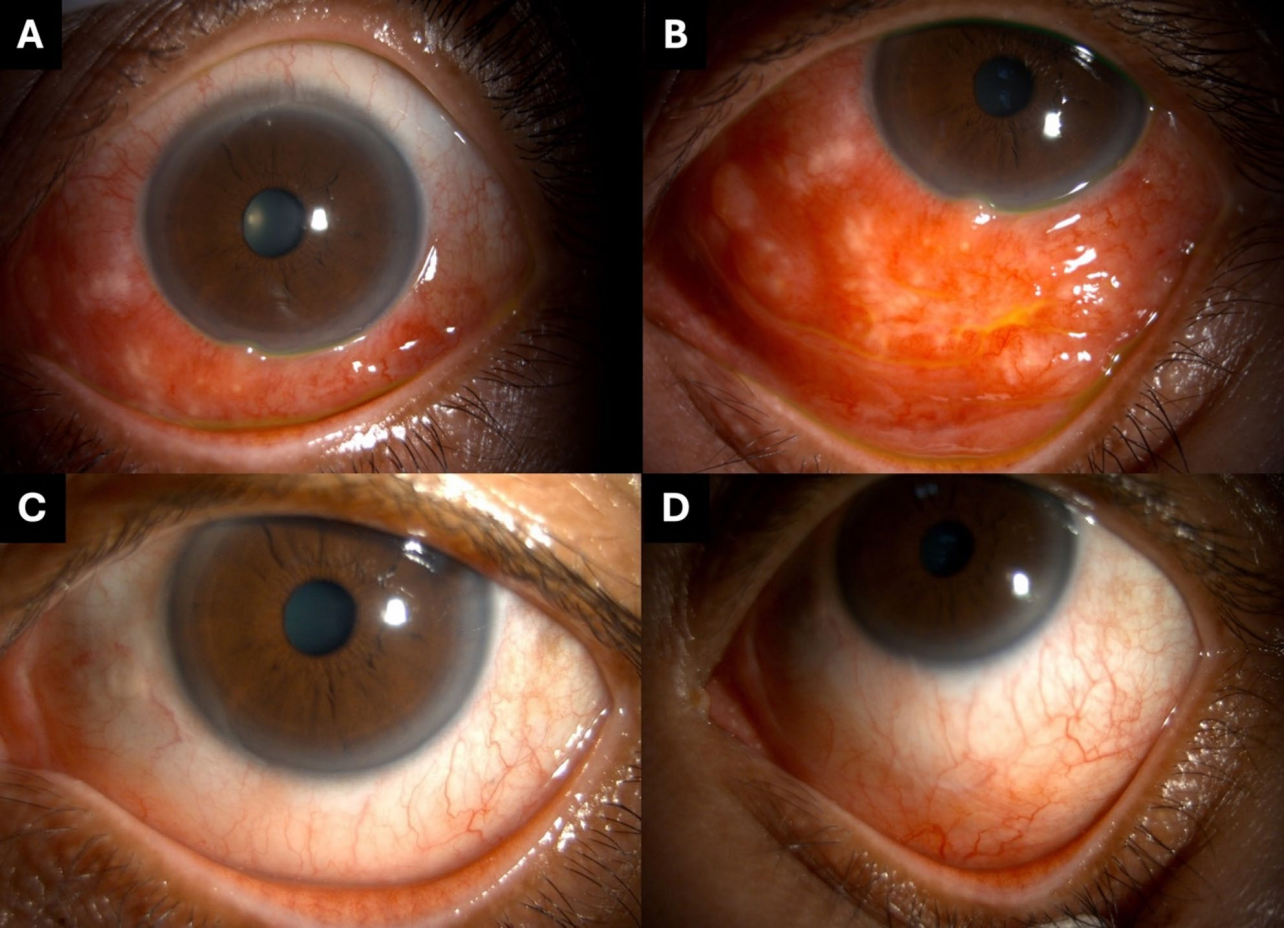
Slit-lamp photographs of Case 1. (**A**, **B**) At presentation, showing a giant papillary reaction involving the inferior bulbar and tarsal conjunctiva, extending to the inferior fornix of the left eye. (**C**, **D**) Three months after treatment, with marked reduction in lesion size and resolution of conjunctival inflammation

A 56-year-old female labourer presented with a two-month history of left eye irritation and redness, without discharge. She had no systemic illness or ocular trauma. Slit-lamp examination revealed multiple giant conjunctival nodular reactions with discrete whitish pinpoint lesions, involving the inferior bulbar and tarsal conjunctiva, extending to the inferior fornix of the left eye (Fig. 2A, B). The initial impression was giant papillary conjunctivitis. A conjunctival biopsy was performed. Routine diagnostic workup showed negative bacterial and acid-fast staining, but fungal culture from a conjunctival biopsy yielded *Sporothrix schenckii*. Histopathology revealed multinucleated giant cells and mixed acute-chronic inflammation. The patient was treated with topical amphotericin B (four times daily) and oral itraconazole (200 mg/day). After six weeks, the lesion significantly decreased in size and topical treatment was discontinued. Oral antifungal therapy was continued for a total of four months. At the final follow-up, residual conjunctival concretions remained as persistent lesions without active inflammation.

### Case 2

**Fig. 3:**
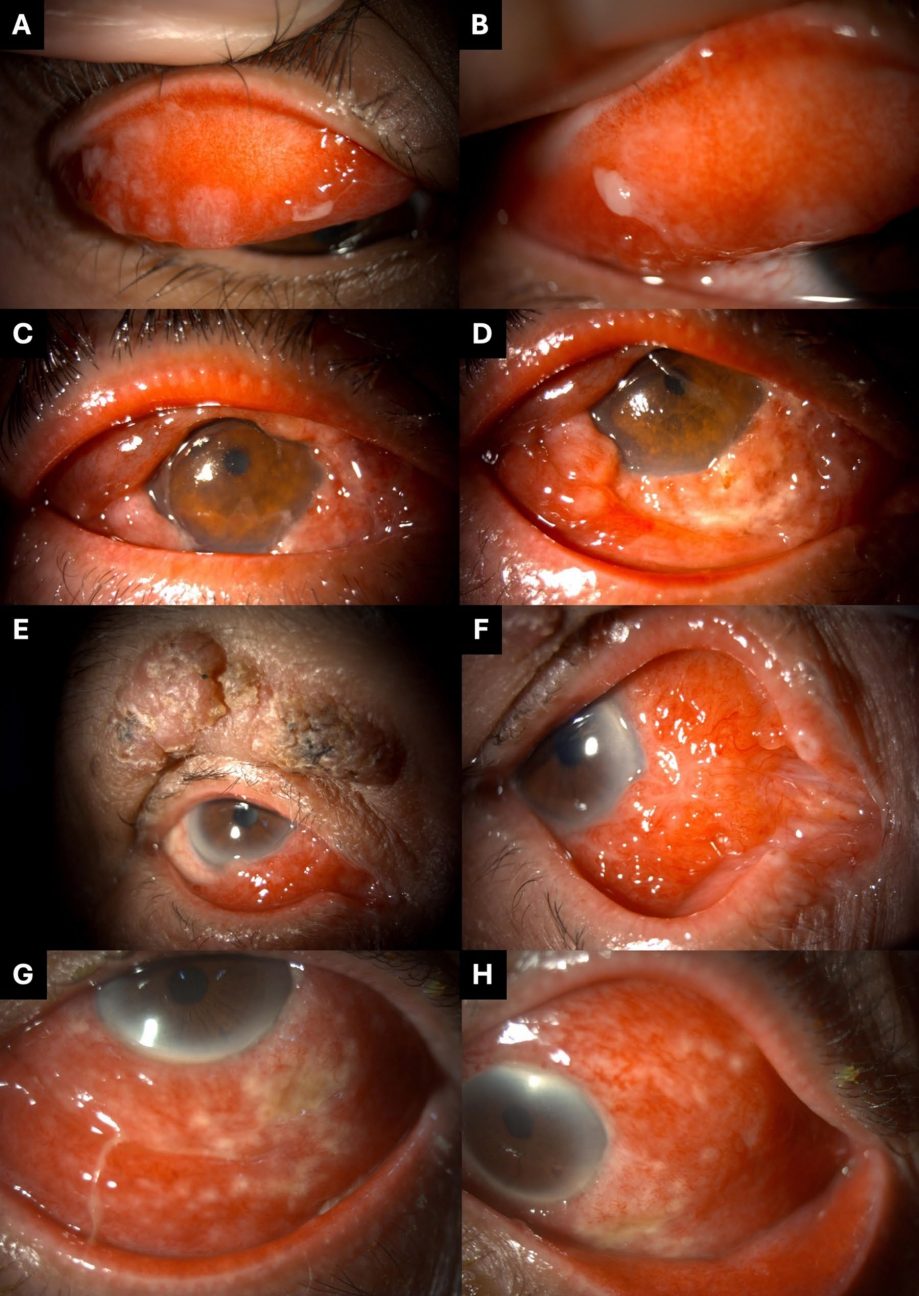
Clinical images of Cases 2–5, demonstrating the varied ocular presentations of sporotrichosis. (**A**, **B**) Case 2: Multiple sub-tarsal nodules with overlying whitish membrane. (**C**, **D**) Case 3: Nodular, yellow-white conjunctival masses on the superior and inferior bulbar surfaces. (**E**, **F**) Case 4: Granulomatous lesion on the upper eyelid with concurrent mass in the inferior conjunctival fornix. (**G**, **H**) Case 5: Follicular conjunctivitis with whitish necrotic nodules and granulomatous changes in the bulbar conjunctiva and lower fornix

A 22-year-old woman reported bilateral eye redness for two weeks. She had no history of systemic illness, ocular trauma, or exposure to animals or soil. Clinical findings included multiple pale, flat-topped sub-tarsal nodules in the superior conjunctiva, with a surrounding follicular reaction and a conjunctival membrane in the right eye (Fig. 3A, B). Scanty mucopurulent discharge was observed, and cervical lymphadenopathy was noted. Culture from conjunctival biopsy was positive for *Sporothrix schenckii*. Histopathology showed chronic granulomatous inflammation and dot-like organisms on GMS and PAS stains. Initial treatment included topical terramycin ointment twice daily and topical 1.5% levofloxacin four times daily for three weeks while awaiting diagnostic confirmation. After the diagnosis was confirmed, the patient was switched to topical amphotericin B and oral itraconazole (400 mg/day for 2 weeks, then reduced to 200 mg/day). The right eye lesion resolved completely, but a new small nodule developed in the inferior conjunctiva of the left eye. After one month of antifungal therapy, the patient showed partial improvement but was lost to follow-up.

### Case 3

A 72-year-old male labourer with hypertension presented with a two-week history of right eye pain, redness, and nodular masses. He was referred from another hospital with suspected scleritis. Examination revealed right eyelid swelling, diffuse conjunctival injection, and multiple nodular, yellow-white masses over the bulbar conjunctiva (Fig. 3C, D). No regional lymphadenopathy was observed. Conjunctival biopsy and fungal culture confirmed *Sporothrix schenckii*, with histology showing granulomatous inflammation and Langhans-type giant cells. The patient later disclosed that both his pet cats had recently died of unknown causes. Treatment included topical natamycin and amphotericin B hourly for three weeks, along with oral itraconazole (200 mg/day) for two months. The conjunctival mass regressed significantly but had not completely resolved before the patient was lost to follow-up.

### Case 4

**Fig. 4:**
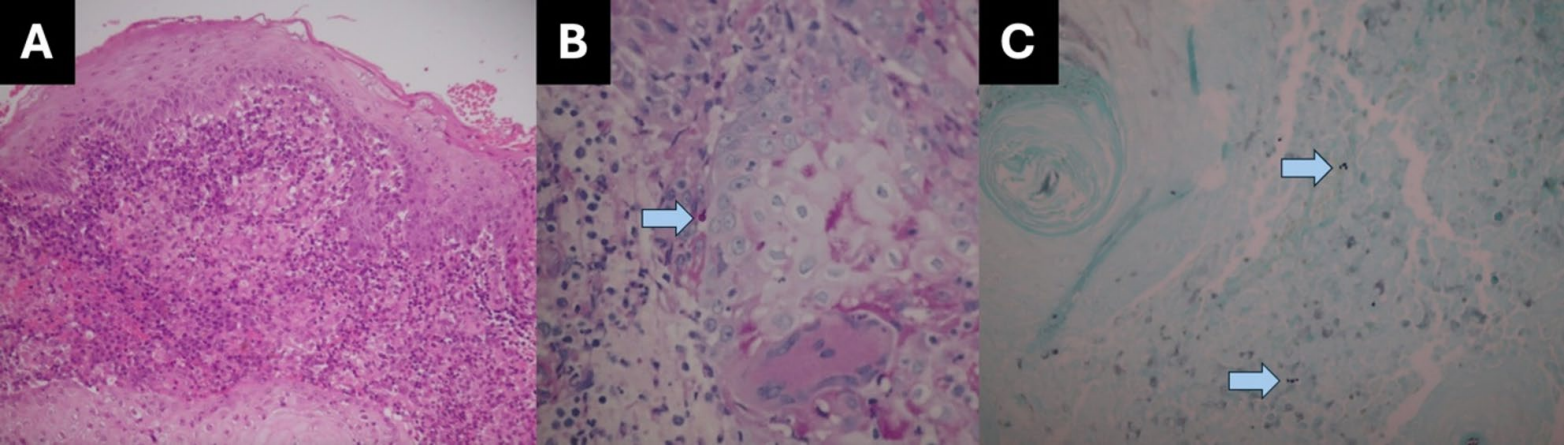
Histopathology images of Case 4. (**A**) Granulomatous inflammation with chronic and few acute inflammatory infiltrates in the dermis of the eyelid skin (H&E, ×200). (**B**) Yeast-forming hyphal elements (PAS, ×600). (**C**) A few groups of yeasts (GMS, ×600)

An 86-year-old retired nun presented with a painless, enlarging right upper eyelid lesion over one month. Her underlying conditions included hypertension and dyslipidaemia. She had a history of being scratched by a stray cat but could not recall the exact timing. Examination revealed a well-defined granulomatous lesion with surrounding eczematous skin on the RUL (Fig. 3E). The initial diagnosis was chalazion. One month later, a conjunctival mass with bumpy, jelly-like surface, was noted in the inferior fornix without surrounding conjunctival inflammation (Fig. 3E, F). Conjunctival biopsy and fungal culture confirmed *Sporothrix schenckii.* Histopathologic findings are shown in Fig. 4, demonstrating granulomatous inflammation with yeast-forming hyphal elements highlighted on PAS and GMS stains. Treatment included topical terramycin twice daily, 0.5% moxifloxacin four times daily, and oral itraconazole (200 mg/day). The lesion decreased in size, but complete resolution was not achieved before she was lost to follow-up after three months.

### Case 5

**Fig. 5:**
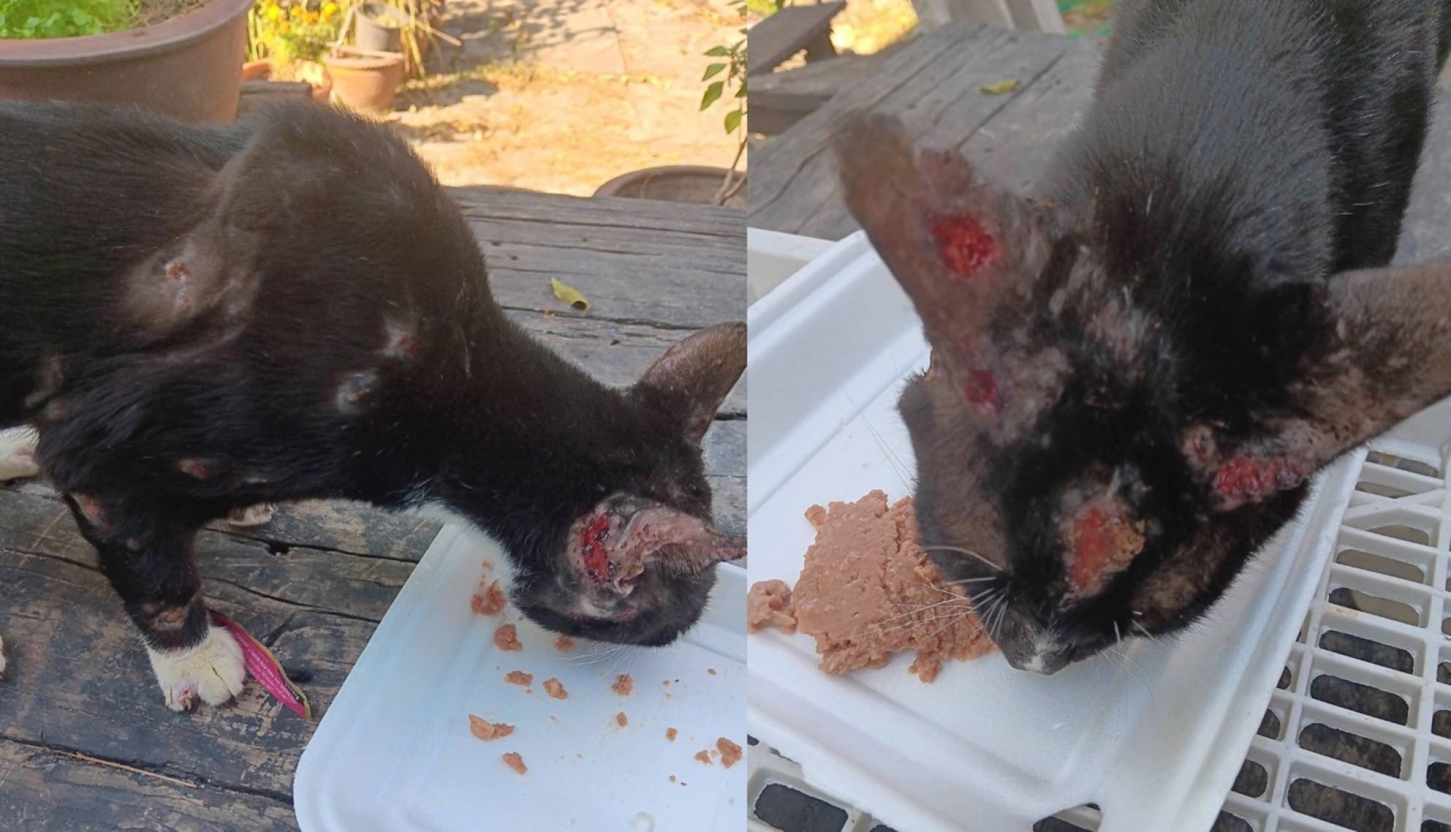
Photographs of the household cat owned by Case 5, showing multiple ulcerative and crusted skin lesions on the ears, face, and limbs, consistent with cutaneous sporotrichosis

A 65-year-old male with a history of diabetes mellitus (DM) and dyslipidemia (DLP) presented with a three-week history of chronic conjunctivitis in the left eye. He denied any recent ocular trauma or animal scratches but reported close contact with a household cat that had multiple ulcerative skin lesions suggestive of a fungal infection (Fig. 5). Examination revealed raised, firm conjunctival nodules with a granulomatous appearance and mild mucous discharge (Fig. 3G, H). A conjunctival swab culture was positive for *Sporothrix schenckii*. Treatment included topical amphotericin B (4–6 times daily) and oral itraconazole (200 mg/day) for two months. The conjunctival granuloma partially regressed before referral to another hospital for continued care.

The key clinical characteristics, diagnostic findings, treatments, and outcomes of all five institutional cases are summarised in Table 1. Outcomes were categorized as complete resolution, partial resolution, persistent lesion, or lost to follow-up. Complete resolution was defined as restoration of the affected area to normal tissue appearance without residual lesion or inflammation.

### Systematic review

A total of 12 studies (7 case reports and 5 case series) published between 2018 and 2024 were included, comprising 23 cases of ocular sporotrichosis from Southeast Asia. One case involving isolated nasal mucosal sporotrichosis was excluded. The selection process is summarized in the PRISMA flow diagram (Fig. 1).

### Patient characteristics and risk factors

Most cases (21 cases, 91.3%) were reported from Malaysia and the remainder from Thailand. The median age was 32 years (IQR 22.5–55.0), with a predominance of female patients (78.3%). The disease was typically unilateral (21 cases, 91.3%). A history of direct or indirect cat contact was documented in 17 cases (73.9%).

### Clinical manifestations

Most patients presented with granulomatous conjunctivitis, often with additional findings such as palpebral or bulbar conjunctival nodules, eyelid swelling, pseudomembrane, or regional lymphadenopathy. The inferior conjunctiva was the most frequently affected anatomical site. All cases were localised to the ocular adnexa with no systemic dissemination.

### Diagnostic methods

Microbiological confirmation of *Sporothrix schenckii* was achieved in 20 cases (87%), most commonly by fungal culture from conjunctival tissue or discharge (*n* = 17), followed by PCR detection (*n* = 2) and histopathologic identification (*n* = 1). The remaining three cases were diagnosed clinically based on characteristic features and favorable response to antifungal therapy. Histopathology was performed in 21 cases (91%), typically revealing granulomatous inflammation, though fungal elements were not always visualized.

### Treatment and outcomes

Systemic antifungal therapy was administered in 21 of 23 patients (91.3%), most commonly with oral itraconazole (18 cases, 78.3%), at 200–600 mg/day. Intravenous amphotericin B was used initially in 2 cases before transitioning to oral itraconazole. Oral fluconazole was prescribed in 3 cases from a single case series. Two patients did not receive systemic therapy. One was treated with topical antifungals alone, and the other received none.

Topical antifungal therapy was used in 6 cases (26.1%), These included fluconazole and amphotericin B, administered either alone or alongside systemic therapy. Intralesional amphotericin B was used in one case as an augmentation to improve local drug delivery. Intravitreal amphotericin B was administered in one case of *Sporothrix* endophthalmitis.

Adjunctive topical antibiotics or corticosteroids were reported in several cases, particularly during the early treatment prior to microbiologic confirmation. Clinical outcomes were favourable in all cases, with complete or near-complete resolution of ocular inflammation. One patient developed persistent limbal stem cell deficiency (LSCD) as a long-term complication, despite microbiological clearance. No systemic dissemination, recurrence, or serious antifungal-related adverse events were reported.

### Comparative findings between institutional case series and published cases

The institutional and published cases shared similar clinical profiles, risk factors, and treatment approaches, though several distinctions were noted.

Among the five institutional cases, the median age was 65 years, compared to 32 years in the 23 published cases, indicating an older affected population. Both groups showed a predominance of females and unilateral conjunctival disease, typically involving the inferior bulbar or tarsal regions.

Cat contact was the primary exposure risk in both series, reported in 60% of institutional and 74% of published cases, highlighting the importance of zoonotic transmission. However, direct proof of cat-to-human transmission was rarely obtained, except in a case by Reinprayoon et al., which demonstrated 100% genetic homology between isolates from a patient and her cat [3]. Environmental exposure was reported in two published cases only, and immunocompromised status was noted in two patients from the literature but in none of the institutional cases.

Regarding diagnosis, all institutional cases underwent histopathology and/or fungal culture, with all showing positive culture results. This is comparable to the 87% culture positivity rate among published cases. A clinical diagnosis without microbiological confirmation was made three cases from the literature.

Treatment protocols were consistent, with all patients receiving oral itraconazole. Topical antifungal therapy was more frequently used as an adjunct in institutional cases. Outcomes were favourable among patients with follow-up, though interpretation was limited by incomplete follow-up in several cases. One literature patient developed persistent LSCD, while no severe complications occurred among institutional cases.

### Quality assessment

All included studies were appraised using the JBI Critical Appraisal Checklists for Case Reports and Case Series [10]. Among the seven case reports, all studies clearly reported patient demographics, clinical history, diagnosis, treatment, and outcomes. Two studies (28.6%) did not report adverse events and were rated “No” for that item, while the remainder explicitly mentioned either observed or absent complications. Overall, all case reports were of good methodological quality.

However, none of the five case series clearly reported whether participants were enrolled consecutively, resulting in an “Unclear” rating for this criterion. Statistical analysis was generally not applicable due to the descriptive nature of the data. Despite these limitations, all studies were judged to be of acceptable quality and were included in the analysis. Detailed item-level results are provided in Supplementary Tables S3 and S4.

**Table 1:** Clinical characteristics, diagnosis, and outcomes of five institutional cases of ocular sporotrichosis

Case	Age/Sex	Affected Eye	Risk Factors	Clinical Findings	Culture results	Histopathology	Topical Treatment	Oral Treatment	Treatment Duration	Response
1	56/F	Left	None	Giant papillae on inferior bulbar and tarsal conjunctiva	Positive for *Sporothrix schenckii*	Granulomatous inflammation	Amphotericin B	Itraconazole 200 mg/day	4 months	Persistent lesions (inactive)
2	22/F	Right, then Left	None	Subtarsal nodules, follicular conjunctivitis, membrane formation	Positive for *Sporothrix schenckii*	Granulomatous inflammation, dot-like organisms (GMS, PAS)	Terramycin, 0.5% Levofloxacin, Amphotericin B	Itraconazole 400 → 200 mg/day	1 month (lost to follow-up)	Partial response before lost to follow-up
3	72/M	Right	Cat exposure	Bulbar conjunctival nodules, eyelid swelling	Positive for *Sporothrix schenckii*	Granulomatous inflammation	Natamycin, Amphotericin B	Itraconazole 200 mg/day	2 months (lost to follow-up)	Partial response before lost to follow-up
4	86/F	Right	Cat scratch	Granulomatous lesion on upper lid and inferior fornix	Positive for *Sporothrix schenckii*	Granulomatous inflammation	Terramycin, 0.5% Moxifloxacin	Itraconazole 200 mg/day	3 months (lost to follow-up)	Partial response before lost to follow-up
5	65/M	Left	Cats and dogs exposure	Conjunctival nodules, chronic conjunctivitis	Positive for *Sporothrix schenckii*	Not performed	Amphotericin B	Itraconazole 200 mg/day	2 months (referred)	Partial response before referral

Abbreviations: M, male; F, female

## Discussion

This study presents five new cases of ocular sporotrichosis from a tertiary referral centre in Thailand and integrates them with 23 previously reported cases across Southeast Asia. One culture-negative case with compatible features was classified as *“suspected ocular sporotrichosis”* and presented separately in Supplementary Appendix 1. Although limited by the small sample size, this represents the first documented case series of ocular sporotrichosis in Thailand. It provides valuable regional data that, together with the systematic review, strengthen recognition of this emerging zoonotic infection in Southeast Asia. In contrast to reports from China, Peru, Brazil, and the United States, where eyelid or intraocular involvement is more common, ocular sporotrichosis in Southeast Asia predominantly affects the conjunctiva [6]. This pattern may reflect regional differences in species distribution and transmission routes, particularly cat-associated zoonosis in Thailand.

Recent reports from Malaysia and Thailand have drawn attention to ocular sporotrichosis as an increasingly recognised clinical presentation of *Sporothrix* infection [3, 7, 11–19]. While the cutaneous and lymphocutaneous forms remain the most prevalent globally, conjunctival and adnexal involvement is being reported with growing frequency [2, 20, 21]. Cat exposure is the principal risk factor in endemic areas [2, 21], and many patients report close contact with infected cats without scratches or bites, suggesting mucosal or self-inoculation routes. Several published reports similarly describe conjunctival sporotrichosis following cat exposure in the absence of direct trauma [3, 12, 13, 18, 19]. Although cats are the most frequently implicated source, rare cases of transmission from dogs have also been identified, expanding the potential range of zoonotic exposure [22]. These trends highlight the need for closer collaboration between medical and veterinary professionals to improve awareness and prevention.

Ocular sporotrichosis frequently mimics common ocular surface diseases, leading to delayed diagnosis, particularly when there is no history of trauma. The typical presentation is unilateral chronic conjunctivitis with yellowish conjunctival granulomas, mucopurulent discharge, and regional lymphadenopathy consistent with Parinaud oculoglandular syndrome [2, 21]. In our observations, two distinct clinical patterns of conjunctival sporotrichosis were noted. Bulbar lesions typically appeared as clustered nodular elevations with whitish, pinhead-sized deposits, whereas tarsal lesions were flat-topped with a rough surface. These differences may reflect the influence of eyelid coverage on lesion morphology. Although corneal involvement was not observed in this study, *Sporothrix* species have been reported to cause keratitis, as demonstrated by Morrison et al. [23], highlighting their potential to infect multiple ocular structures. Less common presentations, such as eyelid lesions and dacryocystitis, particularly in children, may lead to chronic obstruction or cutaneous fistula formation [2, 21, 24–26]. The absence of systemic symptoms or skin lesions in immunocompetent patients further complicates timely recognition, highlighting the importance of clinical suspicion in endemic settings.

Diagnosis relies on clinical suspicion and laboratory confirmation. Diagnostic challenges are shared with other rare fungal pathogens of the ocular surface, where reliance on conventional microscopy or culture can be time-consuming or yield false negatives [27]. Recent reviews emphasize that molecular and point-of-care assays, such as PCR and lateral flow tests, can facilitate earlier diagnosis [28]. Serologic tests such as ELISA are helpful when culture is not feasible. Specific antibody subclasses (IgG2, IgG, IgA, and IgA1) have shown good diagnostic performance, but false-negative results can occur in mucosal forms, early infection, or immunosuppressed patients [29]. A lateral flow assay (Anti-Sporo LFA) offers rapid point-of-care testing with high sensitivity in ocular cases, though it may cross-react with other endemic mycoses and perform poorly in early-stage disease [27]. Combining exposure history with targeted testing is therefore essential in endemic regions. In our setting, there is no standardized guideline for conjunctival swab or biopsy and decisions depend on clinical judgment. Conjunctival swabs are performed when infection is atypical or unresponsive to empirical therapy, whereas biopsy is reserved for histopathologic confirmation or when larger tissue is required. As culture may take up to three weeks, empirical antifungal therapy is usually initiated once suspicion is high. Suspicion increases further when multiple patients present with similar findings within a short period, and treatment is subsequently modified based on microbiological results. Fungal culture should ideally be obtained from discharge or directly from the biopsied lesion. Kongwattananon et al. reported a case of bilateral granulomatous panuveitis due to *Sporothrix* in a patient with polycythaemia vera, underscoring the potential for intraocular spread [30]. 

Oral itraconazole is the recommended first-line therapy for cutaneous sporotrichosis, including presumed ocular involvement, typically 200 mg/day for 3 to 6 months [31]. Although the guidelines do not specifically address ocular disease, published case series support its use in conjunctival and adnexal involvement. Oral itraconazole is usually initiated at 100 mg/day for at least 90 days, with dosage increased in cases of suboptimal response, and antifungal eye drops are generally not used due to insufficient evidence of efficacy [21]. All institutional patients received itraconazole (200–400 mg/day) with partial improvement, though all were lost to follow-up before outcomes could be fully evaluated. No hepatic or systemic adverse effects were observed among patients who completed oral itraconazole therapy. Among those lost to follow-up, no complications were noted at the last visit. However, long-term safety outcomes could not be confirmed due to incomplete follow-up. One suspected case with culture-negative disease achieved spontaneous resolution without antifungal therapy. Several patients had been treated with corticosteroids before diagnosis, which may have delayed recognition or altered disease course. Although corticosteroids can exacerbate fungal infections, their specific role in promoting bulbar conjunctivitis in sporotrichosis remains unclear [21]. Topical corticosteroids should be avoided during active infection and introduced only after clinical improvement under antifungal coverage in compliant patients.

These findings underscore the need for clinical awareness. Chronic unilateral conjunctivitis in patients with cat exposure should raise suspicion for sporotrichosis, and fungal culture or biopsy should be performed early. Corticosteroids must be withheld until infection is excluded [2, 12]. In parallel, public health efforts to reduce zoonotic transmission through veterinary care and community awareness are increasingly important [19, 22]. The emergence of other uncommon ocular fungal pathogens, such as *Macrophomina phaseolina*, further highlights an expanding spectrum of plant-associated pathogens capable of causing eye infections, reinforcing the need for continued clinical and laboratory awareness [32]. 

This study advances understanding of ocular sporotrichosis in Southeast Asia by integrating new institutional data with a regional systematic review. Limitations include the retrospective design, small case number, and loss to follow-up. The systematic review is constrained by the heterogeneous study designs (mainly case reports and small case series), the absence of meta-analytic or subgroup analyses, and the lack of formal certainty assessment. Despite these constraints, this analysis provides a comprehensive overview of the regional experience rather than quantitative analysis. Further prospective studies with species-level identification and standardised treatment protocols are needed to clarify strain-specific differences in presentation and outcomes.

## Conclusion

Ocular sporotrichosis is an emerging infection in Southeast Asia, most often linked to zoonotic transmission from domestic cats. This study, integrating institutional cases with a regional systematic review, underscores its diverse clinical presentations, diagnostic challenges, and favorable response to itraconazole therapy. Early recognition and timely antifungal treatment are crucial to prevent complications. Greater awareness among clinicians, along with improved access to diagnostic tools and public health measures targeting animal reservoirs, will be essential to controlling its spread in endemic regions.

A sixth culture-negative case with compatible features is described separately in Supplementary Appendix 1 as a suspected case.

## Supplementary Information

Below is the link to the electronic supplementary material.

Supplementary Material 1

Supplementary Material 2

Supplementary Material 3

Supplementary Material 4

Supplementary Material 5

Supplementary Material 6

## Data Availability

The datasets generated and/or analysed during the current study are available from the corresponding author on reasonable request.

## Abbreviations

CKD: Chronic kidney disease
DM: Diabetes mellitus
DLP: Dyslipidemia
IRB: Institutional Review Board
ITS: Internal transcribed spacer
LSCD: Limbal stem cell deficiency
PCR: Polymerase chain reaction
RUL: Right upper lid
