# Supplementary material for: Emerging zoonotic ocular sporotrichosis in southeast Asia: a case series from Thailand and systematic review of regional reports
Source: J Ophthalmic Inflamm Infect. 2026 Feb 24;16:12. doi: 10.1186/s12348-025-00565-8 (PMC13035977; doi:10.1186/s12348-025-00565-8)
Supplement: Supplementary file 1 — Supplementary Material 1 [file 12348_2025_565_MOESM1_ESM.docx]

**Supplementary Appendix 1.** Suspected Ocular Sporotrichosis (Case 6)

**Patient summary**

A 75-year-old woman with CKD stage 2, diabetes, hypertension, and a history of coronary bypass surgery presented with six weeks of right eye irritation. She had no prior ocular trauma but reported petting several stray cats near her home. Examination revealed multiple nodules in the inferior bulbar conjunctiva and fornix with surrounding conjunctival injection (Figure 3H). Although microbiological testing from biopsy was negative, histopathology showed chronic granulomatous inflammation. A clinical diagnosis of ocular sporotrichosis was made based on characteristic findings. Treatment included topical terramycin eye ointment once daily and topical 0.5% moxifloxacin four times daily. The lesion gradually decreased in size and completely resolved within two months without recurrence at the final follow-up.

**Interpretation**
 Given the characteristic clinical presentation, exposure to stray cats, and granulomatous inflammation despite negative fungal culture, this case was classified as *suspected ocular sporotrichosis*. It is presented separately to demonstrate the diagnostic challenges that may occur in endemic regions when culture results are inconclusive.


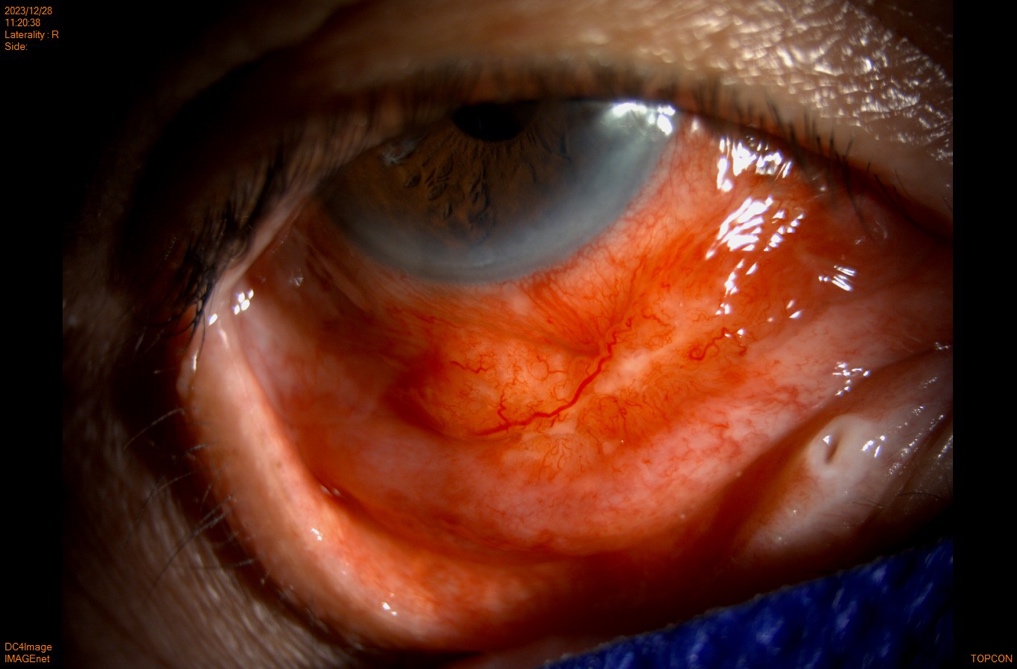


Case 6: Multiple nodular lesions at the inferior bulbar conjunctiva and fornix.
