# Supplementary material for: Emerging zoonotic ocular sporotrichosis in southeast Asia: a case series from Thailand and systematic review of regional reports
Source: J Ophthalmic Inflamm Infect. 2026 Feb 24;16:12. doi: 10.1186/s12348-025-00565-8 (PMC13035977; doi:10.1186/s12348-025-00565-8)

**Supplementary Figure S1A**. Stacked Bar Chart Summary of JBI Appraisal Responses Across Case Reports on Ocular Sporotrichosis


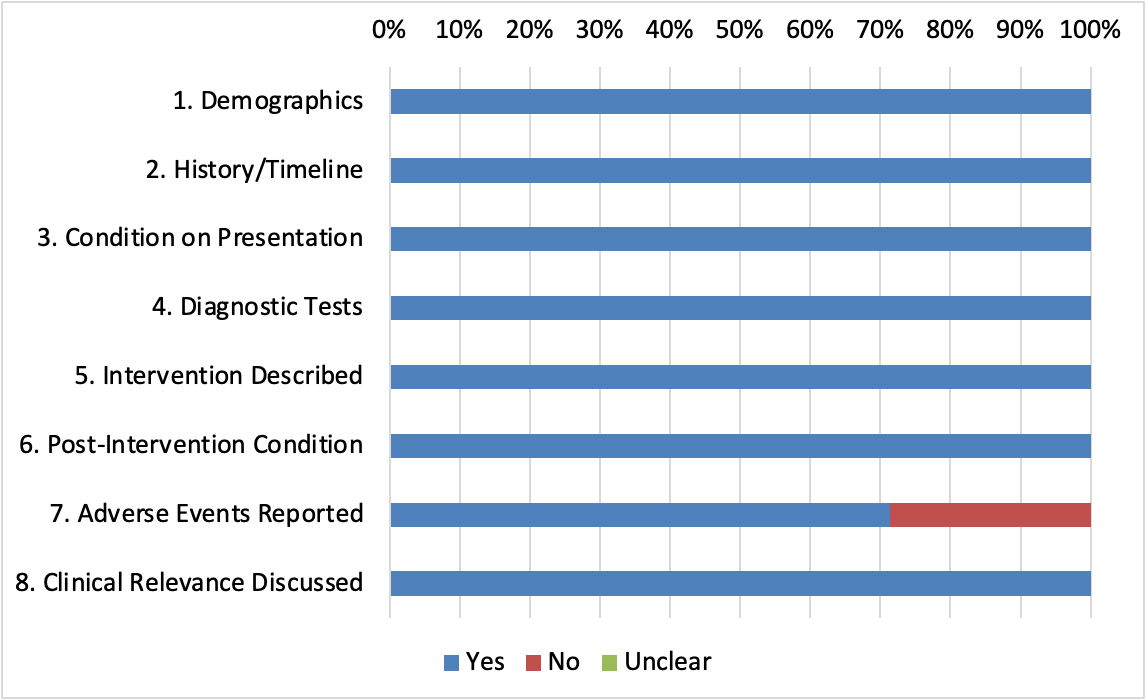


**Supplementary Figure S1B**. Stacked Bar Chart Summary of JBI Appraisal Responses Across Case Series on Ocular Sporotrichosis


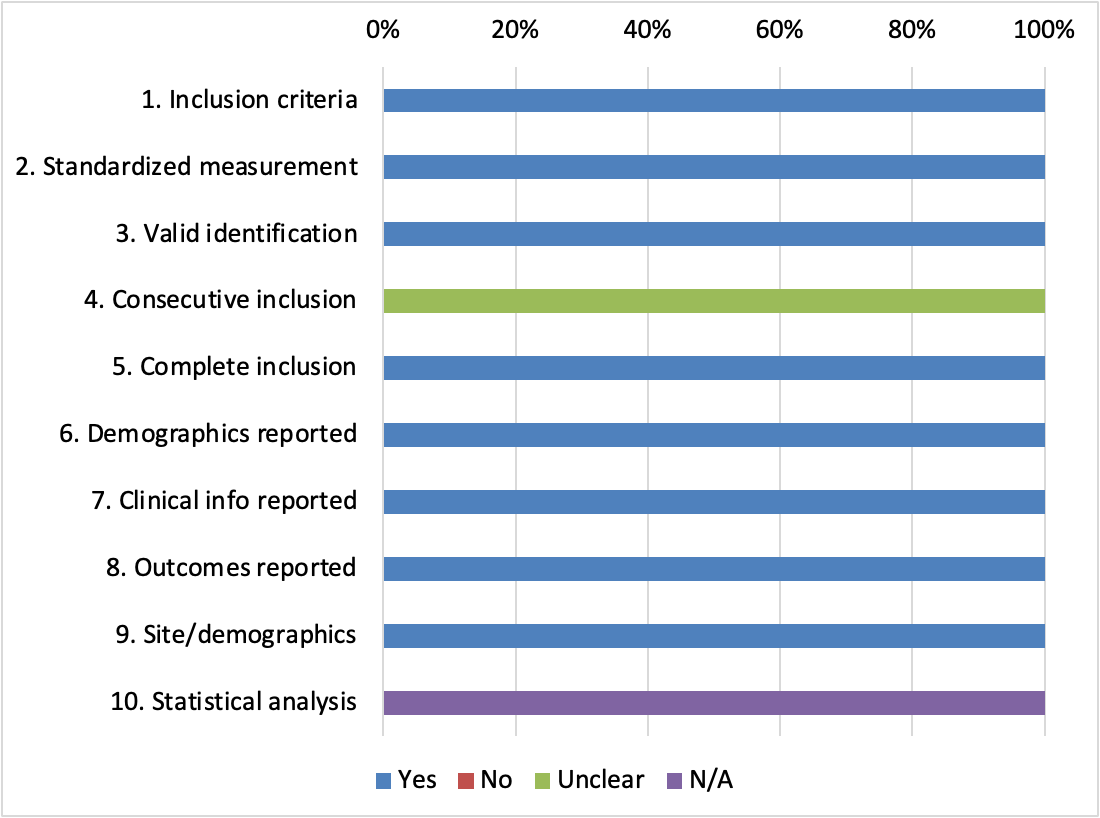

Supplement: Supplementary file 2 — Supplementary Material 2 [file 12348_2025_565_MOESM2_ESM.docx]
