# Supplementary material for: Emerging zoonotic ocular sporotrichosis in southeast Asia: a case series from Thailand and systematic review of regional reports
Source: J Ophthalmic Inflamm Infect. 2026 Feb 24;16:12. doi: 10.1186/s12348-025-00565-8 (PMC13035977; doi:10.1186/s12348-025-00565-8)
Supplement: Supplementary file 3 — Supplementary Material 3 [file 12348_2025_565_MOESM3_ESM.docx]

**Supplementary Table S4**. Quality Assessment of Case Series Using the Joanna Briggs Institute (JBI) Critical Appraisal Checklist

|  | **1. Clear inclusion criteria** | **2. Standardized measurement across participants** | **3. Valid identification methods for condition** | **4. Consecutive inclusion of participants** | **5. Complete inclusion of participants** | **6. Clear reporting of demographics** | **7. Clinical information clearly reported** | **8. Outcomes clearly reported** | **9. Site(s) and participant demographics reported** | **10. Appropriate statistical analysis** | **Overall Appraisal** |
| --- | --- | --- | --- | --- | --- | --- | --- | --- | --- | --- | --- |
| **Ahmad-Fauzi et al., 2022** | Yes | Yes | Yes | Unclear | Yes | Yes | Yes | Yes | Yes | N/A | Included |
| **Mohd Rasidin et al., 2022** | Yes | Yes | Yes | Unclear | Yes | Yes | Yes | Yes | Yes | N/A | Included |
| **Yeow et al., 2023** | Yes | Yes | Yes | Unclear | Yes | Yes | Yes | Yes | Yes | N/A | Included |
| **Tan et al., 2024** | Yes | Yes | Yes | Unclear | Yes | Yes | Yes | Yes | Yes | N/A | Included |
| **Param Jit Singh et al., 2024** | Yes | Yes | Yes | Unclear | Yes | Yes | Yes | Yes | Yes | N/A | Included |
