# Supplementary material for: Emerging zoonotic ocular sporotrichosis in southeast Asia: a case series from Thailand and systematic review of regional reports
Source: J Ophthalmic Inflamm Infect. 2026 Feb 24;16:12. doi: 10.1186/s12348-025-00565-8 (PMC13035977; doi:10.1186/s12348-025-00565-8)
Supplement: Supplementary file 4 — Supplementary Material 4 [file 12348_2025_565_MOESM4_ESM.docx]

**Supplementary Table S1**. Eligibility Criteria for Inclusion in the Systematic Review.

| Category | Inclusion Criteria | Exclusion Criteria |
| --- | --- | --- |
| Study type | Original studies of any design (e.g., case reports, case series, observational studies, cohort studies, cross-sectional studies, clinical trials) reporting at least one human case of ocular sporotrichosis | Animal studies, laboratory experiments, reviews, editorials, conference abstracts without primary data |
| Population | Patients of any age, sex, or ethnicity | Animal studies, in vitro or laboratory-based studies |
| Geographic scope | Studies conducted within countries of Southeast Asia, including Brunei, Cambodia, Indonesia, Laos, Malaysia, Myanmar, the Philippines, Singapore, Thailand, Timor-Leste, and Vietnam. | Studies conducted outside Southeast Asia. |
| Ocular involvement | Any form of ocular or periocular manifestation, including conjunctival, eyelid, lacrimal, or intraocular disease | Studies without ocular or ophthalmic involvement |
| Availability | - | Full-text not available for review |
