# Supplementary material for: Emerging zoonotic ocular sporotrichosis in southeast Asia: a case series from Thailand and systematic review of regional reports
Source: J Ophthalmic Inflamm Infect. 2026 Feb 24;16:12. doi: 10.1186/s12348-025-00565-8 (PMC13035977; doi:10.1186/s12348-025-00565-8)
Supplement: Supplementary file 5 — Supplementary Material 5 [file 12348_2025_565_MOESM5_ESM.docx]

**Supplementary Table S3**. Quality Assessment of Case Reports Using the Joanna Briggs Institute (JBI) Critical Appraisal Checklist

|  | **1. Clear patient demographics** | **2. Clear patient history/timeline** | **3. Clinical condition on presentation** | **4. Diagnostic tests and results** | **5. Intervention(s) or treatment clearly described** | **6. Post-intervention clinical condition described** | **7. Adverse events or complications reported** | **8. Takeaway lessons or clinical relevance discussed** | **Overall Appraisal** |
| --- | --- | --- | --- | --- | --- | --- | --- | --- | --- |
| **Ling et al., 2018** | Yes | Yes | Yes | Yes | Yes | Yes | No | Yes | Included |
| **Lee et al., 2020** | Yes | Yes | Yes | Yes | Yes | Yes | Yes | Yes | Included |
| **Reinprayoon et al., 2020** | Yes | Yes | Yes | Yes | Yes | Yes | Yes | Yes | Included |
| **Theng et al., 2021** | Yes | Yes | Yes | Yes | Yes | Yes | Yes | Yes | Included |
| **Mohamad et al., 2022** | Yes | Yes | Yes | Yes | Yes | Yes | Yes | Yes | Included |
| **Kongwattananon and Rattanaphong, 2023** | Yes | Yes | Yes | Yes | Yes | Yes | No | Yes | Included |
| **Hing and Zahari, 2024** | Yes | Yes | Yes | Yes | Yes | Yes | Yes | Yes | Included |
