# Supplementary material for: Emerging zoonotic ocular sporotrichosis in southeast Asia: a case series from Thailand and systematic review of regional reports
Source: J Ophthalmic Inflamm Infect. 2026 Feb 24;16:12. doi: 10.1186/s12348-025-00565-8 (PMC13035977; doi:10.1186/s12348-025-00565-8)
Supplement: Supplementary file 6 — Supplementary Material 6 [file 12348_2025_565_MOESM6_ESM.docx]

**Supplementary Table S2**. Detailed Search Strategies and Results from Each Database (Search completed on March 20, 2025)

| Database | Search syntax | Results |
| --- | --- | --- |
| PubMed | ("ocular"[Text Word] OR "eye"[Text Word] OR "conjunctiva*"[Text Word] OR "ophthalm*"[Text Word] OR "orbit*"[Text Word] OR "cornea*"[Text Word] OR "uveitis"[Text Word] OR "scleritis"[Text Word] OR "keratitis"[Text Word]) AND ("sporothrix"[MeSH Terms] OR "sporotrichosis"[MeSH Terms] OR ("sporothrix"[Text Word] OR "sporothricosis"[Text Word] OR "sporotricosis"[Text Word])) | 101 |
| Scopus | TITLE-ABS-KEY("ocular" OR "eye" OR "conjunctiva*" OR "ophthalm*" OR "orbit*" OR "cornea*" OR "uveitis" OR "scleritis" OR "keratitis") AND TITLE-ABS-KEY("sporothrix" OR "sporotrichosis" OR "sporotricosis") | 193 |
| MEDLINE via Ovid | ("ocular" or "eye" or "conjunctiva*" or "ophthalm*" or "orbit*" or "cornea*" or "uveitis" or "scleritis" or "keratitis").mp. and (("sporothrix" or "sporotrichosis" or "sporotricosis").mp. or (exp "sporothrix"/ or exp "sporotrichosis"/)) | 111 |
| EMBASE | ('ocular':ta,ab,kw OR 'eye':ta,ab,kw OR 'conjunctiva*':ta,ab,kw OR 'ophthalm*':ta,ab,kw OR 'orbit*':ta,ab,kw OR 'cornea*':ta,ab,kw OR 'uveitis':ta,ab,kw OR 'scleritis':ta,ab,kw OR 'keratitis':ta,ab,kw) AND ('sporothrix':ta,ab,kw OR 'sporotrichosis':ta,ab,kw OR 'sporotricosis':ta,ab,kw) | 102 |
